# Spinal dementia: Don’t miss it, it’s treatable

**DOI:** 10.1007/s00234-024-03425-9

**Published:** 2024-07-10

**Authors:** Horst Urbach, A El Rahal, K Wolf, C Zander, T Demerath, F Volz, J Beck, N Lützen

**Affiliations:** 1 Department of Neuroradiology, Medical Center, Faculty of Medicine, University Medical Center Freiburg, Breisacher Str. 64, 79106 Freiburg, Germany; 2 Department of Neurosurgery, Medical Center, Faculty of Medicine, University of Freiburg, Freiburg, Germany

**Keywords:** Dementia, Frontotemporal brain sagging syndrome, Spontaneous intracranial hypotension, Infratentorial hemosiderosis – CT myelography

## Abstract

**Background & purpose:**

Around 5% of dementia patients have a treatable cause. To estimate the prevalence of two rare diseases, in which the treatable cause is at the spinal level.

**Methods:**

A radiology information system was searched using the terms CT myelography and the operation and classification system (OPS) code 3-241. The clinical charts of these patients were reviewed to identify patients with a significant cognitive decline.

**Results:**

Among 205 patients with spontaneous intracranial hypotension (SIH) and proven CSF leaks we identified five patients with a so-called frontotemporal brain sagging syndrome: Four of those had CSF venous fistulas and significantly improved by occluding them either by surgery or transvenous embolization. Another 11 patients had infratentorial hemosiderosis and hearing problems and ataxia as guiding symptoms. Some cognitive decline was present in at least two of them. Ten patients had ventral dural tears in the thoracic spine and one patient a lateral dural tear at C2/3 respectively. Eight patients showed some improvement after surgery.

**Discussion:**

It is mandatory to study the (thoracic) spine in cognitively impaired patients with brain sagging and/ or infratentorial hemosiderosis on MRI. We propose the term spinal dementia to draw attention to this region, which in turn is evaluated with dynamic digital subtraction and CT myelography.

Whether patients with dementia should receive a MRI scan it still is a matter of debate. Several guidelines recommend acquiring a structural MRI scan in patients with dementia. As appropriate clinical trials are lacking, this recommendation has the level of an expert opinion only (Oxford CEBM level 5) [1]. It is unethical to perform randomized clinical trials in patients with dementia acknowledging that a small percentage (around 5%) of patients suffer from treatable causes of dementia [2, 3]. Common treatable causes are normal pressure hydrocephalus and chronic subdural hematomas, diseases which may be identified also on CT. However, there are treatable causes requiring a more sophisticated imaging work-up – some of those (e.g. CSF venous fistulas CVF) have been discovered in the last few years only [4].

Frontotemporal brain sagging syndrome and infratentorial hemosiderosis are two rare, but treatable causes of dementia - they are typically identified with MRI scans of the head. The underlying cause however, is at the spinal level and once these diseases have been identified the entire spine must be studied.

Frontotemporal brain sagging syndrome (FTBSS) is a dementia form that - from a clinical point of view - can be mistaken for behavioral-variant frontotemporal dementia (bvFTD). Patients present with insidious personality changes, poor judgment, disinhibition, and apathy. One may argue that orthostatic headache is not a feature of bvFTD, however the headache in patients with a frontotemporal brain sagging syndrome is often either absent or stands in the background [5–9]. The clinical similarity between bvFTD characterized by a fronto-temporal atrophy and the frontotemporal brain sagging syndrome with a severe brain sagging is not well understood - it could be related to obstruction of the venous outflow and associated swelling of the diencephalon [8]. In contrast to classic SIH it is noteworthy that in the frontotemporal brain sagging syndrome either no CSF leak or CSF venous fistulas have been identified as underlying causes [9–13].

In infratentorial hemosiderosis caused by repeated subarachnoid hemorrhages and the interaction of cytotoxic hemosiderin with glial tissue dementia is a late symptom. Bilateral sensorineural hearing, ataxia, and pyramidal signs predominate. However, due to the slow progress in a time frame of 10 years or more hearing loss and ataxia may be related to ageing processes and if the underlying cause is not treated symptoms progress and dementia ultimately occurs.

Here, we sought to determine the prevalence of frontotemporal brain sagging syndrome and infratentorial hemosiderosis by analyzing the referral of patients to a tertiary SIH center. We analyzed clinical presentations, imaging findings, and outcomes following treatment.

## Methods

### Patients

A radiology information system was searched using the terms CT-Myelography and the OPS code 3-241. The search was restricted to the study period 1.1.2019-30.6.2023 as the imaging work-up was different before. In 2018, we introduced dynamic digital subtraction myelography (DSM) and CT myelography (CTM) in prone and/ or lateral decubitus position [14]. In brief, when patients show epidural fluid on heavily T2-weighted spine images (so called spinal longitudinal extradural CSF collection SLEC) we expect a ventral or lateral dural tear from which CSF has oozed into the epidural space. These patients are studied with prone or lateral decubitus DSM and CTM. When patients do not show epidural fluid (SLEC-negative) patients could have a CSF venous fistula and are studied with lateral decubitus DSM followed by lateral decubitus CTM. The lumbar needle is kept in place and the second half of contrast (another 7 ml) is injected after acquisition of the scout images [14, 15].

**Fig. 1:**
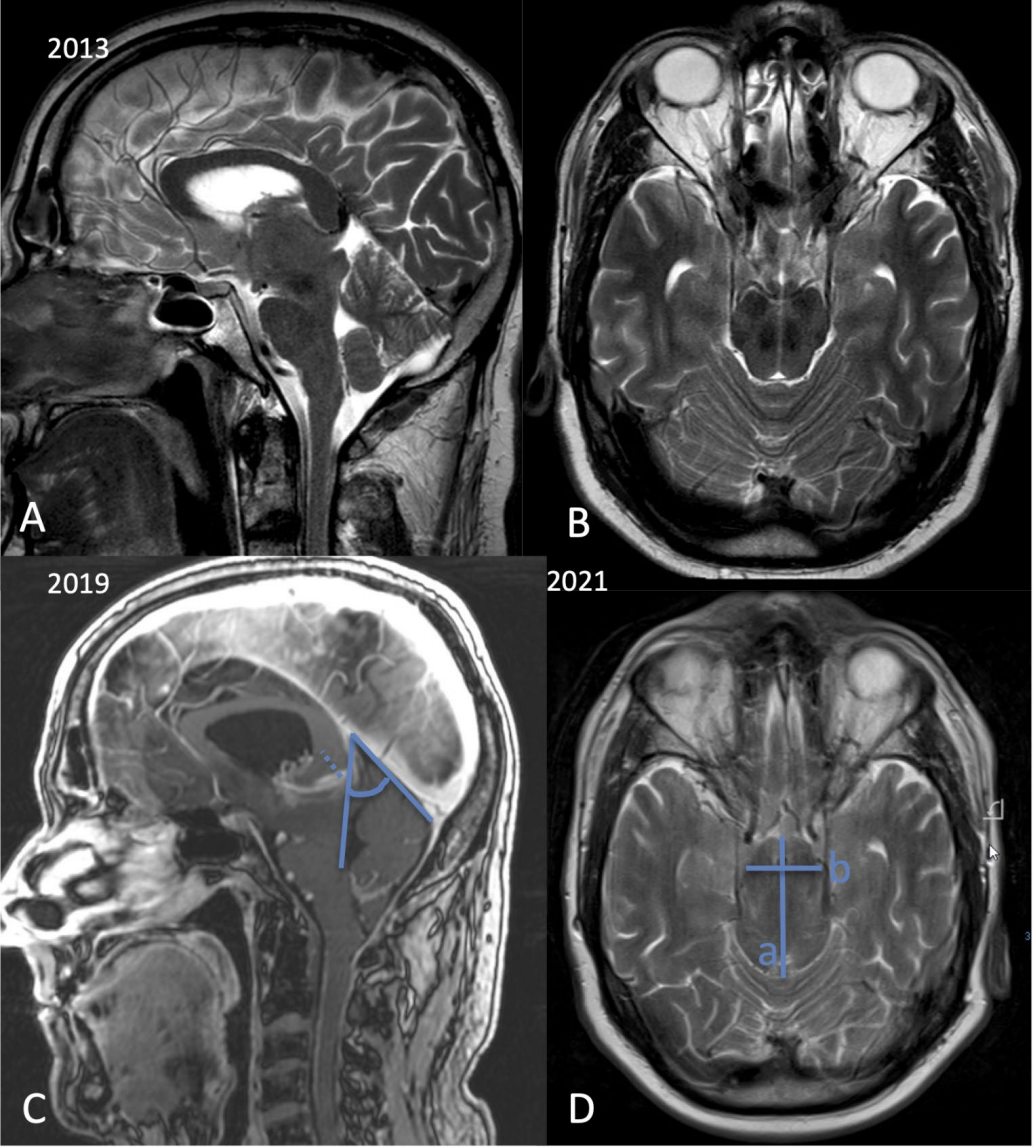
A 57-year-old man presented with apathy, loss of interest, gait ataxia, and tremor. Over at least 6 years symptoms had progressed so that he was only sitting looking television the whole day. Sagittal (**A**) T2-weighted and contrast enhanced MPRAGE images (**C**) show a small angle between the vein of Galen and the straight sinus (C: angle). The vein of Galen is elongated as indicated by the distance from the confluence of the internal cerebral veins and the exit of the vein of Galen in the straight sinus (C: dashed arrow). Axial T2-weighted images (**B**, **D**) show a marked and progressive midbrain elongation indicated by a long a.p. diameter of the midbrain and a higher sag ratio a/b

Patients with proven CSF leaks were retrieved from the patient’s charts. In these patients, two neuroradiologists reviewed MRI images, determined the Bern score and calculated measures indicating diencephalic and midbrain deformation: The maximum antero-posterior and bipeduncular distances were documented and a sag ratio was calculated by dividing the maximum antero-posterior distance by the.

maximum bipeduncular distance [16] (Fig. 1). The angle between the vein of Galen and the straight sinus was measured and a parallel line indicating the length of the vein of Galen was drawn from the internal cerebral vein confluence to the exit of the vein of Galen into the straight sinus (Fig. 1). Measures were performed before and after treatment.

Moreover, SWI, T2* or - if not present - b0 diffusion weighted (DWI) images were reviewed to identify patients with infratentorial hemosiderosis.

## Results

**Fig. 2:**
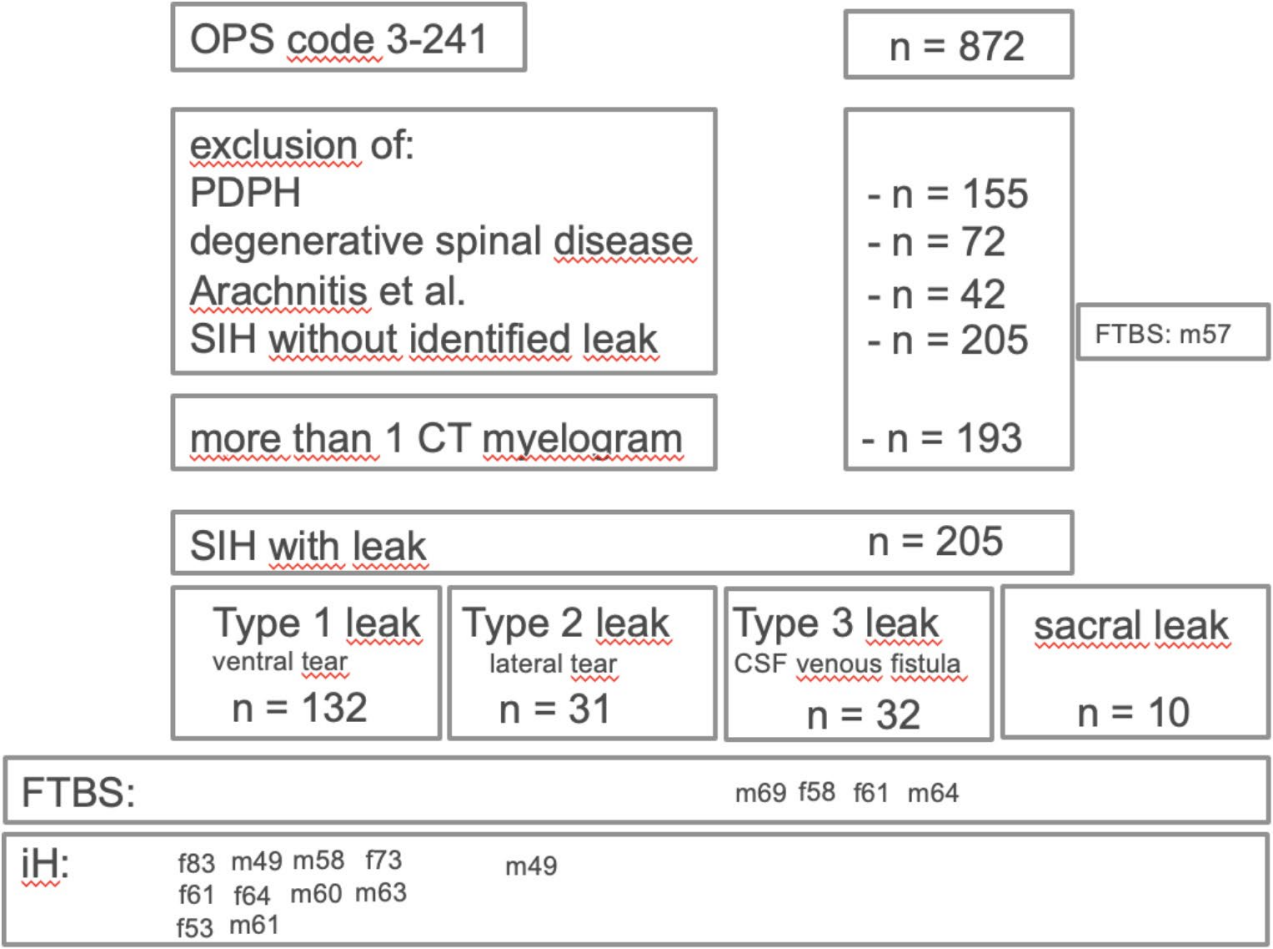
Study flowchart

Patient’s identification process is detailed in Fig. 2. Clinical characteristics are summarized in Tables 1 and 2.

**Table 1:** Frontotemporal brain sagging syndrome (FTBSS)

Sex, age	Clinical symptoms	symptom onset - diagnosis	Imaging	Treatment & Outcome
m, 57	2013: apathy, loss of interest, gait ataxia, tremor	6 years	2021: Sag ratio 1.7, vein of Galen-angle 51°, -length 13.7 mm, mamillo-pontine distance 0 mm 2021: DSM, CTM: normal	2019, 2021: several epidural blood patches 2021: no changes on MRI or clinically
f, 58*	2009: orthostatic headache, vomiting, speech difficulties, gait ataxia 2014: not able to work any longer	5 years	2021: Sag ratio 1.1, vein of Galen-angle 39°, -length 16 mm, mamillo-pontine distance 0 mm 2021: DSM, CTM: CVF T8/9 left, 9/10 right 2022: DSM, CTM: several de novo CVF	2020: surgical ligation CVF T8/9 left, transvenous embolization CVF T9/10 right 2022: Sag ratio 0.96, vein of Galen-angle 74°, -length 8 mm, mamillo-pontine distance 0 mm clinical improvement, still not able to work
m, 69	2015: apathy, sleepiness, dysphagia Large venous malformation of the right side of the thorax	4 years	2019: Sag ratio 1.4, vein of Galen-angle 59°, -length 12.1 mm, mamillo-pontine distance 1 mm 2019: DSM, CTM: CVF T8/9 right	2019: surgical ligation CVF T8/9 right, 2019: Sag ratio 0.89, vein of Galen-angle 76°, -length 5 mm, mamillo-pontine distance 2 mm marked clinical improvement
f, 61*	2020: apathy, forgetfulness, not able to handle the household	1 year	2021: sag ratio 1.07, vein of Galen-angle 39°, -length 27 mm, mamillo-pontine distance 0 mm DSM, CTM: CVF T8/9 right	2021: transvenous embolization CVF T8/9 right 2022: sag ratio 0.98, vein of Galen-angle 51°, -length 22 mm, mamillo-pontine distance 3 mm no complaints, able to work again
m, 64	2022: disinhibition, not able to work as teacher. Large venous malformation of the right side of the body.	1 year	2023: sag ratio 1.17, vein of Galen-angle 62°, -length 13 mm, mamillo-pontine distance 2 mm DSM, CTM: CVF T2/3 right	2023: transvenous embolization CVF T2/3 right 2024: sag ratio 0.9, vein of Galen-angle 93°, -length 10 mm, mamillo-pontine distance 3 mm marked clinical improvement

CSF venous fistulas CVF

**Fig. 3:**
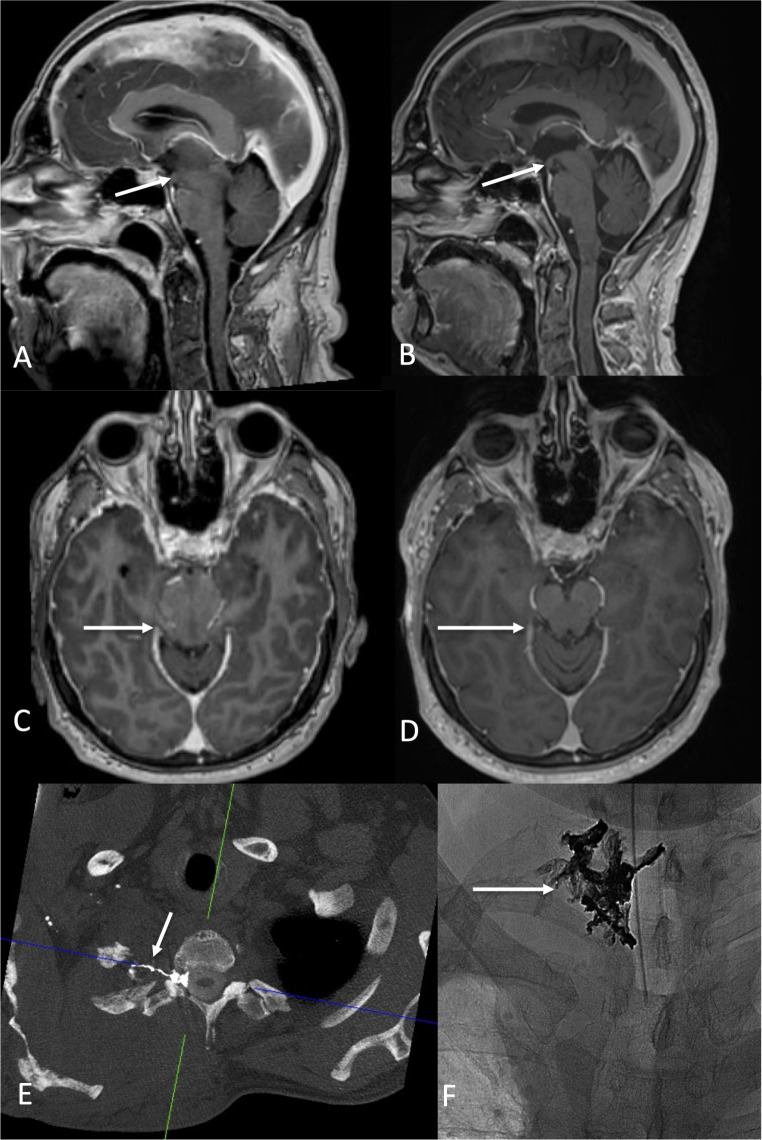
A 64-year-old man presented with disinhibition, dizziness and logorhoea so that he was not able to work as a teacher any longer. MRI shows severe midbrain sagging with an extremely low mamillo-pontine distance (**A**: arrow) and an elongated midbrain (**B**: arrow). Dynamic CT myelography discloses a CSF venous fistula at the right-sided T2 nerve root (**C**: arrow). Following transvenous embolization (**F**: arrow points to the Onyx cast), MRI changes are regredient (**D**, **E**: arrows) and the patient reported on marked improvement

We identified five patients with a frontotemporal brain sagging syndrome and a disease history of up to more than six years. Bern score in all patients were 7 to 9. Four of those patients had one or more CSF venous fistula as underlying cause and significantly benefitted from either surgical clipping (*n* = 2) and/ or transvenous embolization (*n* = 4) (Table 1) (Fig. 2). Parallel to the clinical improvement, MRI changes were regressive: The sag ratios decreased from 1.19 +- 0.14 to 0.93 +- 0.04, the vein of Galen angle increased from 49.8 +- 12.5 to 73.5 +- 17.3°, and length of the vein of Galen decreased from 17 +- 6.9 to 11.25 +- 7.46 mm, respectively (Figs. 1 and 3).

In one patient, we were not able to identify a CSF leak and applied several high volumes epidural blood patches without behavioral or MRI changes of the patient.

**Fig. 4:**
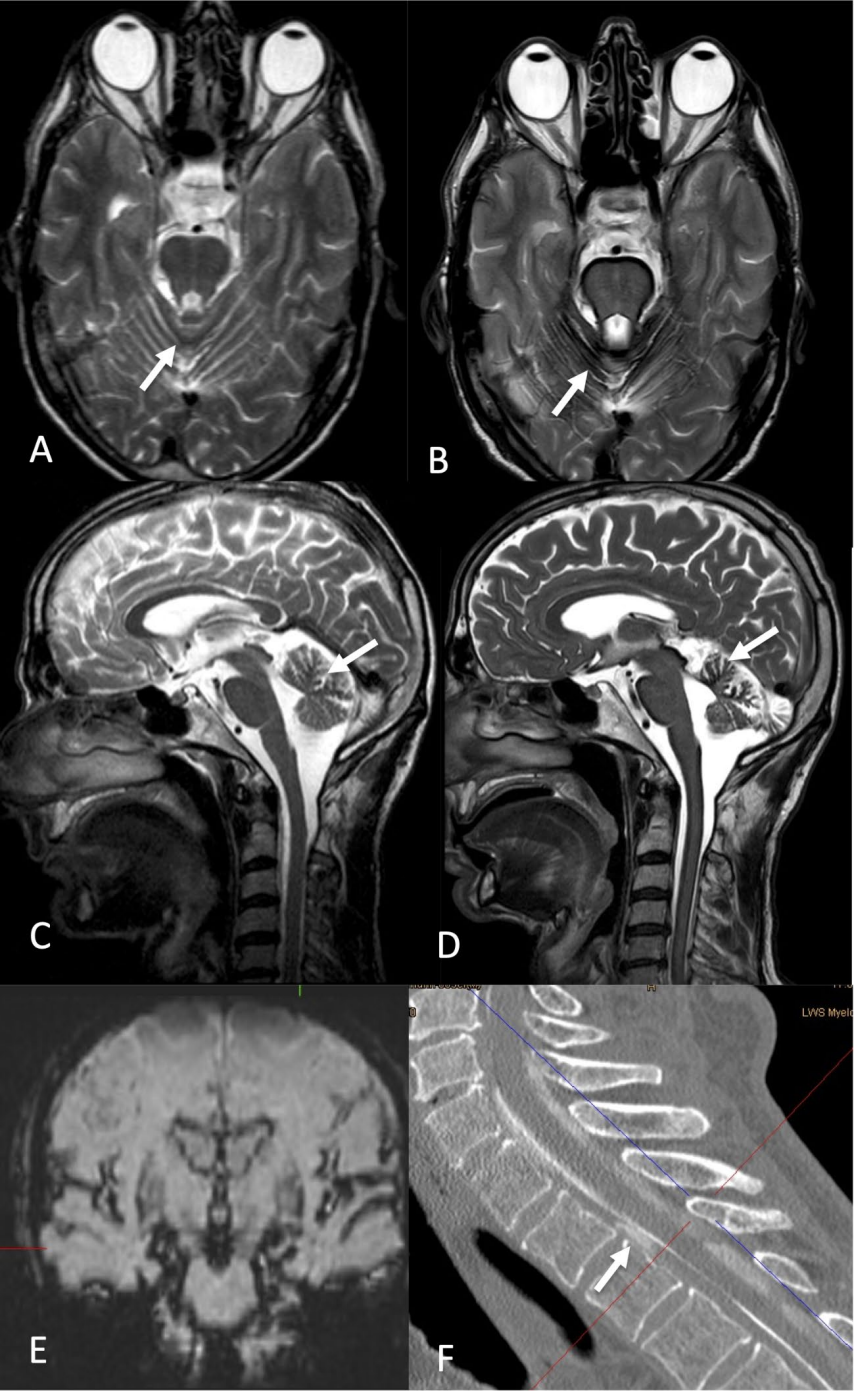
A 61-year-old man complained of vertigo progressive over some years. He could not walk stairs, had hearing difficulties, depression, and was unable to work. Initial MRI 2 years after onset of orthostatic headaches was considered normal but shows subtle infratentorial hemosiderosis (**A**, **C**: arrow). Six years later, hemosiderosis and cerebellar atrophy had progressed (**B**, **D**: arrow). A coronal SWI sequence shows the full extent of hemosiderosis (**E**). Work-up with dynamic DSM and CTM disclosed a ventral leak at T2/3 (**F**: arrow)

**Fig. 5:**
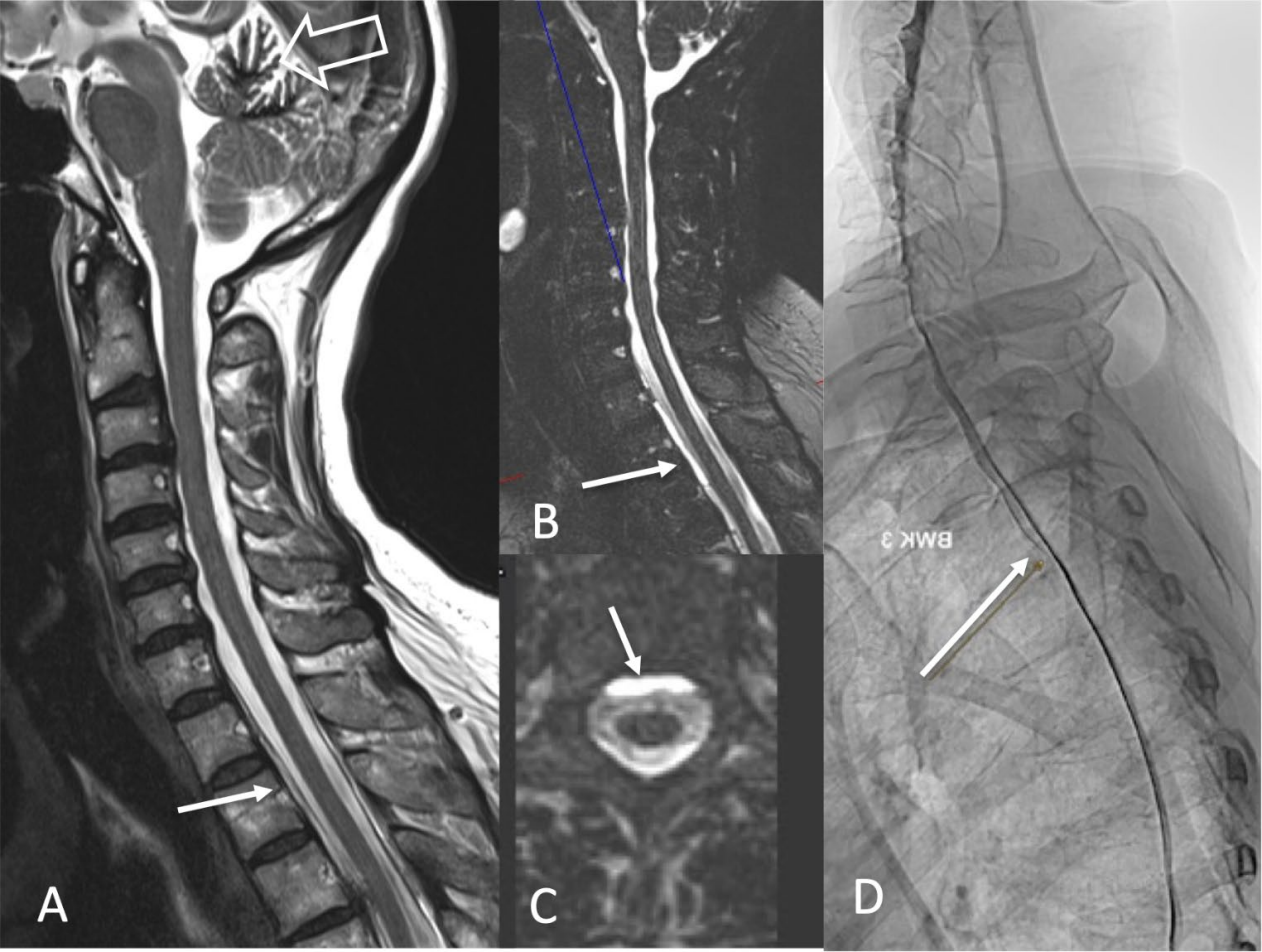
A 54-year-old woman complained of headache, head pressure ear ringing, blurry vision, gait disorder, and cognitive “slowing”. Sagittal T2-weighted MRI of the cervical spine shows infratentorial hemosiderosis with hemosiderin in the foliae of the upper vermis (**A**: hollow arrow) and spinal longitudinal epidural fluid (SLEC) (**A**: arrow). A heavily T2-weighted fat-suppressed SPACE sequence allows the calculation of axial images with the majority of the epidural fluid ventral to the spinal cord (**B**, **C**: arrow). Digital subtraction myelography with a high temporal resolution (1 frame/ second) was needed to capture the time point of egress of contrast from the subarachnoid space (**D**: arrow) – here, a non-subtracted image is displayed

We identified another 11 patients with infratentorial hemosiderosis (Table 2) (Figs. 4 and 5). Ten patients had ventral dural tears and one patient a lateral dural tear at C2/3 after a motor bike accident 35 years ago, respectively. Of note are the progressive hemosiderin deposition in the foliae cerebellae of the upper vermis which was shown in six patients who had MRI scans before and the long history of up to 36 years between the putative event and the presentation in an ENT department due to hearing problems or in a Neurology department when ataxia and cognitive decline predominated.

**Table 2:** Infratentorial hemosiderosis

Sex, age	Clinical symptoms	symptomonset– diagnosis	Imaging	Treatment & Outcome
m, 49	2017: vertigo with retroflexion of the head, occasionally ringing in the ears	2 years	2019: MRI: infratentorial hemosiderosis, SLEC 2019: DSM, CTM: lateral leak C3/4 left	No follow up
m, 61	2010: orthostatic headache 2018: progressive gait ataxia, vertigo, unable to walk stairs, hearing difficulties, depression, unable to work	9 years	2010: MRI: infratentorial hemosiderosis, SLEC 2019: MRI: infratentorial hemosiderosis, SLEC 2019: DSM, CTM: ventral leak T2/3	2010: epidural blood patch 2012: surgery CSF fistula C2 right 2019: surgical closure ventral leak T2/3 2020: no headache, no vertigo, ataxia and hearing difficulties unchanged
f, 83	2014: headache, slurred speech, hearing loss, gait ataxia	5 years	2014: MRI: infratentorial hemosiderosis, no spine MRI 2019: MRI: infratentorial hemosiderosis, SLEC 2019: DSM, CTM: ventral leak T2/3	2019: surgical closure ventral leak T2/3 2021: gait unsteadiness and slurred speech improved
m, 49	Orthostatic headaches, neck and back pain, bilateral hearing loss, cochlear implant, double vision	10 years	2015–2019: MRI: infratentorial hemosiderosis, SLEC, 0.85, 84.2° 2019: DSM, CTM: ventral leak T7/8	2019: surgical closure ventral leak T7/8 2020: orthostatic headaches improved, works again
m, 58	Taste and smelling disorder, gait unsteadiness, memory complaints	6 months	2019: MRI: infratentorial hemosiderosis, SLEC, sag ratio 0.85, vein of galen angle 64.1° 2020: DSM, CTM: ventral leak T2/3	2020: surgical closure ventral leak T2/3 2021: alertness and gait unsteadiness improved
f, 73	Gait unsteadiness, bilateral hearing loss, imbalance (wheel chair	36 years	2020: MRI: infratentorial hemosiderosis, SLEC, 0.96, 87.1° 2020: DSM, CTM: ventral leak C7/T1	surgical closure ventral leak C7/T1 orthostatic headaches improved
f, 61	Gait unsteadiness, paraparesis, sits in wheel chair	9 years	2020: MRI: infratentorial hemosiderosis, SLEC 2020: DSM, CTM: ventral leak T2/3	2020: surgical closure ventral leak T2/3 2022: fine motor skills improved, still sitting in wheel chair
f, 64	slurred speech, gait unsteadiness	0 years	2020: MRI: infratentorial hemosiderosis, SLEC 2020: DSM, CTM: ventral leak T2/3	2022: surgical closure ventral leak T2/3 2023: symptoms improved
m, 60	Gait unsteadiness bilateral hearing loss with cochlear implant, lower limb weakness	12 years	2020: MRI: infratentorial hemosiderosis, SLEC 2020: DSM, CTM: ventral leak T3/4	2020: surgical closure ventral leak T3/4 2022 No improvement
m, 73	Hearing problems, CI intended	2 years (?)	2007: MRI: thoracic spine with SLEC 2022: MRI: infratentorial hemosiderosis SLEC 2022: DSM, CT-M: ventral leak T6/7	2022: surgical closure ventral leak T6/7 2023: hearing improved
f, 54	Headache, head pressure ear ringing, blurry vision, gait disorder, cognitive “slowing” (MoCA© 22/30), episode of orthostatic headache in 2013	10 years	2022: MRI: infratentorial hemosiderosis SLEC, sag ratio 0.87, vein of galen angle 73.4° 2023: DSM, CT-M: ventral leak T3/4	2023: surgical closure ventral leak T3/4 2024: persisting SLEC, still complaints, MoCA© 27/30

*Case report of these patients have been published [13, 25]

## Discussion

Frontotemporal brain sagging syndrome (FTBSS) is a cumbersome term linking a clinical syndrome (behavioral variant frontotemporal dementia (bvFTD-)like presentation) and a radiological presentation. MRI is somewhat different from classic SIH with orthostatic headache: The midbrain is more elongated and especially the thalami and midbrain are sagged downwards (Figs. 2 and 3). An extreme midbrain deformation may be accompanied by a bilateral thalamic or midbrain edema, when CSF is lost rapidly and even lead to coma or death [17, 18]. The prevalence of FTBSS in a tertiary SIH center is very low and estimated to less than 2%. While the cause of a frontotemporal brain sagging syndrome was unknown for more than a decade it is now obvious that at least 1/3 of cases are caused by CSF venous fistulas [8, 9]. CVF were first described by Schievink et al. in 2014, when they acquired dynamic digital subtraction myelograms in lateral decubitus position [4]. Recent refinements like resisted inspiration or the incremental use of CT myelography or a second contrast injection in the CT scanner further increased the detection rate [15, 19]. Thus, when patients show severe brain sagging on head scans and no epidural fluid (spinal longitudinal extradural CSF collection SLEC) on spine scans, lateral decubitus myelograms and CT myelograms must follow. This work-up is recommended as CVF can be easily treated surgically, with CT-guided fibrin glue injections, or with transvenous embolization [9, 13, 20, 21].

Dementia is a rare and rather late presentation in patients with infratentorial hemosiderosis. Most patients (around 5% in a tertiary SIH center) present with bilateral sensorineural hearing loss and/or ataxia. However, as these symptoms develop slowly, the underlying cause remains often undetected. It is also not uncommon that patients with infratentorial hemosiderosis had a history of SIH with the key symptom orthostatic headache a long time ago. Patients were sometimes treated with epidural blood patches, improvements of the headaches were interpreted as cure through the blood patches, and the initial presentation with headaches was forgotten. As the great majority of infratentorial hemosiderosis cases is caused by ventral dural tears existing over many years and as these tears do not disappear with epidural blood patches [22], it is mandatory to precisely locate them and to fix them by surgery. The ventral dural tears are suspected when MRI scans of the spine show (ventral) epidural fluid (SLEC) and the slit-like, vertically orientated, often only rice grain-like tears are only found when patients are studied in prone and head-down position [14]. Of note are the facts that the hemosiderin deposits progress over time (Figs. 4 and 5) and that the latency period between an initial event and surgery has an impact on improvement after surgery: Patients with a long latency period (of around > 8 years) are less likely to improve [23].

Limitations: It cannot be excluded that we missed FTBSS or hemosiderosis patients in the group of patients who were transferred with suspected SIH in which SIH was not proven according to ICDH criteria [24]. Only few FTBSS and hemosiderosis patients were formally tested with dementia scores. While the FTBSS patients fulfill clinical dementia criteria with symptoms severe enough to interfere with daily life, hemosiderosis patients show more subtle cognitive changes and are rather limited by hearing and ataxia problems (Fig. 5).

Conclusion: Frontotemporal brain sagging syndrome and infratentorial hemosiderosis are two rare, but treatable diseases with the underlying cause in the spine. We propose to use the term spinal dementia to increase the awareness for studying the spine in these patients.

## Abbreviations

CVF: CSF venous fistula
FTBSS: Frontotemporal brain sagging syndrome
SIH: Spontaneous intracranial hypotension
iH: Infratentorial hemosiderosis
CTM: Dynamic CT myelography
